# Who Benefits From Being an Only Child? A Study of Parent–Child Relationship Among Chinese Junior High School Students

**DOI:** 10.3389/fpsyg.2020.608995

**Published:** 2021-01-08

**Authors:** Yixiao Liu, Quanbao Jiang

**Affiliations:** Institute for Population and Development Studies, School of Public Policy and Administration, Xi’an Jiaotong University, Xi’an, China

**Keywords:** only children, sibship size, birth order, children’s gender, parent–child relationship, China

## Abstract

After more than three decades of implementation, China’s one-child policy has generated a large number of only children. Although extensive research has documented the developmental outcomes of being an only child, research on the parent–child relational quality of the only child is somewhat limited. Using China Education Panel Survey (2014), this study examined whether the only child status was associated with parent–child relationships among Chinese junior high school students. It further explored whether children’s gender moderated the association between the only child status and parent–child relationships. Two-level ordered logit models suggested that only children were more likely to report a close relationship with their mothers and fathers compared to children from multiple-child families (including two-child families). Taking birth order into consideration, we found that, only children were more likely to have close parent–child relationships than firstborns, whereas no significant differences were found between only children and lastborns. Interaction analyses further suggested that the only child advantages were gender-specific: the positive effects of the only child status were stronger for daughters than for sons, that is, daughters benefited more from being only children. Our findings highlight the importance of considering children’s gender and birth order in exploring the only child effects in the Chinese context. Additional analyses about sibling-gender composition indicated female children were more likely to be disadvantaged with the presence of younger brothers, whereas male children benefited more from having older sisters. This reveals that the son preference culture is still deep-rooted in the Chinese multiple-child families.

## Introduction

In 1979, China implemented the highly controversial One-Child Policy (OCP) which required the number of children for each couple to be limited to only one Child (Falbo and Hooper, 2015). Exceptions existed in a few cases. For example, couples who were ethnic minorities, whose first child had disabilities, or whose (from rural areas) first child was a girl could get the chance to have a second child with permission (Li et al., 2015). The One-Child Policy, coupled with the socio-economic development, made China’s fertility sharply fall from 6 in the 1970s to 1.5 in 2010 (Cai, 2013). Although this policy ended on January 1, 2016 and was replaced by a universal Two-Child Policy (Qian and Jin, 2018), the profound impacts of this policy on Chinese society still persist (Chi et al., 2020).

One of the impacts is the generation of large numbers of one-child families. In 2010, the total number of only children rose to 145 million (Wang, 2013). This special group has attracted the attention of many scholars (Chi et al., 2020). A growing body of literature has documented the developmental outcomes of being an only child. Generally speaking, two views exist in academia with regard to the welfare of growing up as an only child (Liu et al., 2010). One view supports the negative side. The notion “being an only child is a disease in itself,” remarked by Fenton (1928), has provided a base for the popular thinking that only children tend to be spoiled by their parents and grandparents (Mancillas, 2006; Liu et al., 2010). This idea argues the adults in the families tend to prioritize the needs of the only child, which could result in adverse developmental outcomes of this child, such as dependence, self-centeredness, and indifference (Roberts and Blanton, 2001; Mancillas, 2006). In addition, because only children have no siblings to interact with, they perhaps lack proper interpersonal skills to efficiently negotiate their relationships with their peers (Downey and Condron, 2004). Based on this idea, the popular media usually referred to Chinese only children as “little emperors” (Fong, 2004; Falbo, 2012).

However, the above popular thinking was deemed a stereotype for only children (Mancillas, 2006) because it was not supported by most empirical studies both in the West (Falbo and Polit, 1986; Mellor, 1990; Falbo, 2012) and in China (Poston and Falbo, 1990; Falbo and Poston, 1993; Guo et al., 2018). Therefore, the other perspective about only children was more positive in its nature: only children tend to be either normal or more advantaged compared to those with siblings in many developmental dimensions (Falbo and Polit, 1986; Polit and Falbo, 1987; Mellor, 1990; Falbo, 2012; Chen and Liu, 2014). In China, studies of only children have focused on a variety of outcomes. Concerning academic outcomes, Chinese children without siblings appear to have higher academic achievements and cognitive abilities than children with siblings (Poston and Falbo, 1990; Falbo and Poston, 1993; Jiao et al., 1996). With regard to psychological outcomes and character features, some studies observed no significant differences between Chinese only children and non-only children (Poston and Falbo, 1990; Guo et al., 2015; Wang et al., 2020), and others reported better outcomes of only children (Liu et al., 2010; Falbo and Hooper, 2015; Guo et al., 2018). In terms of the traditional virtues, research demonstrated that although Chinese only children did not differ from their non-only counterparts in the sense of family obligation or filial piety (Fuligni and Zhang, 2004; Deutsch, 2006), they are more motivated to have higher achievements in order to assume the responsibility supporting their aging parents (Fong, 2002, 2004).

Even though an extensive body of literature has made comparisons between the Chinese only, and non-only children on a variety of developmental outcomes (such as academic, psychological, and behavioral outcomes), only a few studies have focused on the comparison of the parent–child relationships between the two groups. According to Western research, the variations in parent–child relationships could explain the differences in developmental outcomes between only children and non-only children (Falbo and Polit, 1986; Polit and Falbo, 1988; Mellor, 1990; Falbo, 2012). Meta-analyses conducted by Falbo and Polit (1986) suggested that the different developmental outcomes between only children and non-only children is because the former group have a special parent–child relationship characterized by increased parental anxiety and attention (Falbo and Polit, 1986; Falbo, 2012). Specifically, parents of only children tend to be more anxious than their multiple-child counterparts because of their inexperience in rearing children (Falbo and Polit, 1986). In this case, parents of only children would be more careful and responsive in the child-rearing activities than parents of more children, leading to high-quality parent–child relationships (Falbo and Polit, 1986). Further, the high-quality parent–child relationships would encourage children to interact more with their parents, thereby resulting in a stimulating home environment which was beneficial for only children’s developments (Polit and Falbo, 1988). However, such parent–child relational pattern of only children is observed based on Western literature (Falbo, 2012). Whether the parent–child relationships in Chinese families vary with the sibling status? Are Chinese only children more likely to have a close relationship with their parents than their non-only counterparts? Whether the only child effects, if any, differ based on children’s characteristics? This study is designed to answer the above questions.

### The Only Child Status and Parent–Child Relationships in Chinese Families

According to attachment theory, parent–child relationship plays an important role in shaping children’s development (Videon, 2005; Levin et al., 2012; Ma et al., 2020). Studies have consistently documented the significant impacts of relationship with caregivers on children’s developmental outcomes in China and other cultures (Dmitrieva et al., 2004; Chen, 2017; Li et al., 2018; Xu et al., 2019). A harmonious parent–child relationship provides children a sense of security, which is fundamental for their well-being (Li et al., 2018). For example, in a study conducted among Shanghai public school students (age = 15.3 years), children’s attachment to mothers as well as fathers was found to predict their academic engagement (Chen, 2017). Another study using nationally representative data demonstrated that, in addition to academic achievement, parent–child relationships (together with parental presence) also influenced Chinese children’s cognitive and psychological outcomes (Xu et al., 2019).

The nature of parent–child relationships is highly influenced by culture and social structure (Chow and Zhao, 1996). In Chinese families characterized by Confucian culture, parents have greater authority and power in the hierarchical parent–child relationship than their Western counterparts (Chow and Zhao, 1996; Lu and Chang, 2013). Therefore, Chinese children are required to obey their parents on any child-related issues and filial piety is regarded as a necessary virtue a person should have (Chow and Zhao, 1996). However, influenced by the Western culture emphasizing individualism, Chinese parent–child relationship is becoming more egalitarian in recent years (Sun, 2011). Meanwhile, with the development of social economy, the children’s economic value drops while their emotional value increases (Goh and Kuczynski, 2010; Sun, 2011). A child-centered culture has gradually risen in Chinese families (Tsui and Rich, 2002). In this situation, Chinese parents are becoming emotionally closer to their children than before (Tsui and Rich, 2002; Sun, 2011). Therefore, considering the dramatic changes taking place in Chinese families as well as the shifts in parent–child relational pattern in recent years, it is particularly important to gain insight into parent–child relationship in modern Chinese families.

According to family systems theory, many factors determine the quality of parent–child relationship, such as marital relationship of parents (Li et al., 2018). In the present study, we mainly focus on the effects of only child status (family size) on parent–child relationship. A negative association between family size and parent–child relationship is widely reported in the Western literature (Falbo and Polit, 1986). For example, studies of Western families have demonstrated that the parent–child relational quality was higher in one-child families than in larger families (Falbo and Polit, 1986; Falbo, 2012). In Lewis and Feiring (1982)’s study, family members were observed more likely to be involved in conversations including more frequent parent–child discussions during family meals in one-child families than in multiple-child families. Some studies focusing on the comparisons between only children and non-only children also took birth order into consideration (Falbo and Polit, 1986; Mellor, 1990; Falbo, 2012). Meta-analyses of Western literature showed that, although only children have better relationships with their parents than non-only children in general, they are not significantly different from firstborns or children from two-child families (Falbo and Polit, 1986; Falbo, 2012). The Largest differences usually came from the comparisons between only children and children with more than one sibling or children of later born (Haan, 2010; Falbo, 2012). As discussed above, this is because parents of only children, firstborns, or children with only one sibling have greater anxiety about parenting (more responsive to children’s needs) and more attention in child rearing activities (Falbo and Polit, 1986; Falbo, 2012).

The resource dilution model could explain the link between sibship size and parent–child relationships. The term “resource dilution” is first used by Blake (1981) to describe the relationship between family size and the quality of children. Resource dilution model argues that parental resources are not infinite and with the increase in the number of children, the resources invested in each child decrease (Blake, 1981). Parental resources can take many forms, such as those providing a supportive home environment, opportunities to engage with the outside world, and direct treatments, such as attention (Polit and Falbo, 1988; Gibbs et al., 2016). The parent–child relationship is also a kind of parental resource because it is closely related to parental time (attention) spent on children or parent–child interactions: the more time parents devote to their children, the closer the parent–child relationship is (Li et al., 2015).

Although limited, there are still a few studies analyzing how sibship size influences Chinese parent–child relationships. Most of the existing research suggested a more positive parent–child relationship of only children compared to their sibling counterparts. Using data of Beijing schools, Chow and Zhao (1996) showed that parents of only children spent a greater proportion of their leisure and total time on their singleton children than did parents of non-only children. The author also compared other parental resources invested in only children and non-only children and found that the only children were generally in a more advantaged position (Chow and Zhao, 1996). Hao and Feng (2002) used data collected from Hubei Province and found that parents of only children interacted more frequently on both verbal and physical activities with their children than did parents of non-only children. Wei et al. (2016) observed an only child advantage in maternal educational involvement in Chinese families. In a qualitative study by Deutsch (2006), compared to children with siblings, children without siblings were found to be more concerned with the parent–child relationship and have closer emotional bonds with their parents. By analyzing the social behaviors of Beijing kindergarteners, Li et al. (2015) found that non-only children had slightly closer mother–child relationship than did only children. This pattern is not in line with the resource dilution model perhaps because the sampled families were highly selected and the multiple-child families in Beijing had more resources: the mothers of non-only children did not have to work. In this case, non-only children might have more time to interact with their mothers than only children whose mothers working outside the home (Li et al., 2015).

In sum, existing studies were limited and findings were mainly based on regional data. More representative national-scale data are needed to further examine how only children and non-only children are emotionally attached to their parents and whether there are significant differences between the two groups. Western studies have detected the birth-order effects that only children were no different from firstborns but significantly different from laterborns in terms of parent–child relationships (Falbo and Polit, 1986). Does this pattern apply to Chinese children? Studies of Chinese only children failed to do the comparisons between only children and children of different birth order regarding parent–child relationship. Therefore, this study also aims to fill in the research gap by considering the birth order of children.

### The Role of Children’s Gender

Influenced by Confucianism culture, children’s gender plays important role in Chinese parenting strategies. Due to the patriarchal, patrilineal, and patrilocal structure, women are subordinate to men and young women are in the lowest strata of the family hierarchy (Shu, 2004). In this system, daughters are traditionally devalued because they would eventually marry into another family and would have to contribute to that family. Natal families could not see benefits in investing in daughters (Xie, 2013). However, this is not true for sons. Sons are not only expected to support their elderly parents but also responsible for carrying on the family lines (Sun, 2002). Therefore, investments in sons was deemed more rewarding than investments in daughters. As a result, Chinese parenting strategies have been characterized by a son preference for a long time (Guo et al., 2018). The female infanticide in Chinese history is a proof of that (Das Gupta et al., 2003). However, as discussed above, with the implementation of the One-Child Policy and socio-economic development, a child-centered phenomenon is emerging in Chinese families (Tsui and Rich, 2002). By having fewer children or only one child, parents would not show gender preference in their parenting strategies (Tsui and Rich, 2002). Empirical studies have found a narrowing male-favorable gender gap in education (Ye and Wu, 2011) or even a reversed educational gender gap among the Chinese only child group (Lee, 2012). For example, Ye and Wu (2011) found that gender inequality in education among younger cohorts was less prominent than among older cohorts due to the fertility decline in China. This implies that the daughter benefits more from having fewer siblings or being an only child in intra-household resources allocation than does the son (Lee, 2012).

Parent–child relationship is a reflection of emotional and time resources parents invest in children. Therefore, when applying the resource dilution model to analyzing the link between sibship size and parent–child relationship in Chinese families, children’s gender needs to be given special attention (Chu et al., 2007). To the best of our knowledge, little research has examined the role of children’s gender in the association between sibship size and parent–child relationship. To fill in the important research gap, this study will gain an insight into whether the only child advantages (in parent–child relationship), if any, are more prominent among daughters than sons. Previous studies also paid attention to the gender of siblings (Chu et al., 2007; Zheng, 2015; Guo et al., 2018). Due to the strong son preference, having brothers (especially younger brothers) would reduce one’s opportunities in obtaining family resources, whereas having sisters (especially older sisters) would generally improve one’s well-being (Chu et al., 2007; Zheng, 2015). For example, a study in Taiwanese families indicated that parents tended to discontinue the older daughters’ education and further encouraged them to make economic contributions to the whole family and their younger siblings (usually brothers) (Chu et al., 2007). This led to more education of those with older sisters. For the well-being of children, brother(s) presence is an unfavorable factor, while sister(s) presence is a favorable factor (Zheng, 2015). Considering the importance of siblings’ gender, this study also compared only children to children with siblings of different gender.

### The Present Study

This study is designed to explore whether Chinese only children are more advantaged in emotional relationship with their parents compared to non-only children. Meanwhile, we also aim to compare only children with the firstborns, the middleborns, and the lastborns from multiple-child families to identify the birth-order effects. Furthermore, considering the gendered characteristics of family relationships in China, we will analyze whether children’s gender plays a moderating role in the association between the only child status and children’s parent–child relationship. Finally, we will compare only children to children with siblings of different gender (sibling-gender composition) regarding parent–child relationships.

The data used in this study derived from a national survey of school-going adolescents (junior high school students, 48.66% female, age range: 12 – 18; average age = 14.5 years). We used this dataset — China Education Panel Survey (2014) — based on the following reasons. First, adolescence is a period when people are undergoing critical changes in psychological, physical, and social development (Ruhl et al., 2015). Influenced by these changes, during this period, children are more vulnerable to their social relationships with parent–child relationships being the most important. The quality of parent–child relationships during adolescence has been found to influence the adolescents’ developmental outcomes (Ruhl et al., 2015; Chen, 2017; Li et al., 2018; Xu et al., 2019), with the influences persisting well into adulthood and later life (Hair et al., 2008; Raudino et al., 2013). Second, the increased autonomy and shared-decision making with parents during adolescence enable adolescents to be more objective in their evaluations of their relationships with parents (Ruhl et al., 2015). Third, the sampled adolescents in our study had a mean age of 14.5 years at 2014 meaning that they were born around 2000 when the one-child policy had been in force for almost 30 years. The phenomenon of one-child families had become a social norm (Falbo and Hooper, 2015; Falbo, 2018) and a child-centered culture had taken shape in Chinese society. Parenting strategies were thus unique for this generation (the one-child policy began to be relaxed around 2013, see Jiang and Liu, 2016). Therefore, it is interesting to explore the only child effects on parenting strategies for this generation. Lastly, because Chinese culture continues to value education highly (Huang and Gove, 2015), junior high school education, which plays an important role in transitioning to high school education, is emphasized by Chinese parents. Due to the highly competitive nature of attaining entrance to high schools in China, there is much stress placed on junior high school students to prepare for the graduation examination—that allows them to enter high-quality high schools (Wu, 2015). In this process, parents also make their own contributions to their children such as providing harmonious family relationships. Furthermore, a junior high sample is more representative of Chinese adolescents in general because this educational stage is covered by the Nine-Year Compulsory Education (Guo et al., 2019). Many adolescents could not go to high schools due to a lack of family resources (Loyalka et al., 2013). The website of China’s Ministry of Education shows that in 2012, around 98% of primary graduates entered junior high schools, whereas only 88% of junior high graduates entered high schools [MEPRC (Ministry of Education of the People’s Republic of China), 2019]. Based on this, it is important to analyze parent–child relationships among junior high school students.

The following content of the paper is divided into four parts: (1) an introduction of materials and methods used in the study; (2) a report of the results from the descriptive analyses and the multilevel models; (3) a discussion of the empirical findings; (4) a summary of the study.

## Materials and Methods

### Data

We used data from the baseline of China Education Panel Survey (CEPS 2014). CEPS is a nationally representative survey aiming at investigating how individual educational outputs are impacted by family, school, and community. Conducted by Renmin University of China, the data were gathered with a fourth-stage probability sampling design that randomly selected 19,487 students of grade 7 and grade 9 from 438 classes across 112 junior high schools in 28 counties (districts) of mainland China. Students along with their parents (19,487), teachers (438), and school faculty (112) constituted the final survey sample.

Five types of major questionnaires were used in the survey to collect information on students, their parents, homeroom teachers, main subject teachers (Chinese, Math, and English), and school administrators. The student questionnaires were completed by students collectively in the classroom and the parent questionnaires were completed by their corresponding parents or their main caregivers at home (copies of the parent questionnaires were taken home by the students). The study variables in this paper were mainly derived from the student questionnaires. All the survey data were collected using a paper/pencil measure. The data had a response rate of 98.7%.

We merged students’ data and parents’ data and 19,487 parent–child pairs were generated. One hundred and sixty five (0.85%) observations were deleted due to the missing information on dependent variables. In the remaining sample, most of our explanatory variables had a very low level of missing in formation (ranging from 0 to 2.5%) with parental age at birth of the respondent child (around 25% missing) and gender of siblings (around 10% missing) being the exceptions. Apart from parental age at birth and gender of siblings, the missing percentage for the whole sample were 5.35%. To avoid losing too many observations, we created a “missing” category for the variables with high rate of missing information (will elaborate later in the “measure” part). Thus, the final analytical sample was 18,445.

### Measures

#### Dependent Variables

##### Parent–child relationship

Research has measured parent–child relationships in a variety of ways. Some studies employed parental verbal and physical interactions with children, parental control, and prenatal supportiveness through specific and multi-dimensional items to measure parent–child relationships (Pritchett et al., 2011; Li et al., 2015; Chen et al., 2018; Xu et al., 2019; Ma et al., 2020). Others utilized a single and general item measuring parent–child relationships (Videon, 2005; Damsgaard et al., 2014). For example, Videon (2005) operationalized parent–child relationship using a single question: “Overall, are you satisfied with your relationship with your mother (father)?” Damsgaard et al. (2014) employed the question: “how easy is it for you to talk to your mother/father about things that really bother you?” In our study, we employed the later practice: capturing the quality of parent–child relationship with a single general question. Meanwhile, because mothers and fathers tend to play different roles in parenting activities (Liu, 2020), and the child’s development is usually influenced by his/her same-sex parent (Ohannessian, 2012), it is necessary to measure father–child and mother–child relationship separately.

In the present study, parent–child relationships were assessed with one item about each parent. On the student questionnaire, children were asked to rate the relationship with their parents: how is the general relationship with your mother/father? Responses included “not close (2.4% for mother–child relationship and 4.3% for father–child relationship),” “moderate (24.21% for mother–child relationship and 33.28% for father–child relationship),” and “close (73.40% for mother–child relationship and 62.42% for father–child relationship).” We created a three-category ordinal variable for mother–child closeness and father–child closeness (0–2, a higher value indicates closer parent–child relationship), respectively. See Table 1 for the measurements of dependent variables.

**TABLE 1:** Definitions and measurements of the study variables.

**Variable**	**Definition and Measurement**
Mother–child relationship	How is the general relationship between the child and his/her mothers, ordinal variable (0 = not close; 1 = moderate; 2 = close)
Father–child relationship	How is the general relationship between the child and his/her fathers, ordinal variable (0 = not close; 1 = moderate; 2 = close)
Sibship size	Sibship size of the child, 3-category [0 = no sibling (only child), 1 = 1 sibling, 2 = 2 + siblings]
Birth order	Birth order of the child, 4-category [0 = only child, 1 = firstborn (of the multiple-child family), 2 = middleborn (of the multiple-child family), 3 = lastborn (of the multiple-child family)]
Younger bothers	Whether the child has younger brother(s), 4-category [0 = only child, 1 = without younger brother(s) (for child having siblings), 2 = with younger brother(s) (for child having siblings), 3 = younger brothers missing]
Younger sisters	Whether the child has younger sister(s), 4-category [0 = only child, 1 = without younger sister(s) (for child having siblings), 2 = with younger sister(s) (for child having siblings), 3 = younger sisters missing]
Older brothers	Whether the child has older brother(s), 4-category [0 = only child, 1 = without older brother(s) (for child having siblings), 2 = with older brother(s) (for child having siblings), 3 = older brothers missing]
Older sisters	Whether the child has older sister(s), 4-category [0 = only child, 1 = without older sister(s) (for child having siblings), 2 = with older sister(s) (for child having siblings), 3 = older sisters missing]
Total number of children in the family	Total number of children in the family, continuous variable (1–6); those having more than six children were coded as 6 due to the low proportion (<0.4%)
Children’s gender	Gender of the child, dummy variable (0 = son; 1 = daughter)
Grade	Grade of the child, dummy variable (0 = grade 7, 1 = grade 9)
Ethnicity	Whether the child is ethnic minority, dummy variable (0 = Han ethnicity, 1 = ethnic minority)
Cognitive score	The standard score of children’s cognitive abilities, continuous variable (from –2.03 to 2.71)
Academic performance	Self-rated academic performance of the child, continuous variable (from 0 to 4); the higher the score, the better of the performance
Boarding	Whether the child attends a boarding school, dummy variable, (0 = no, 1 = yes)
Parental co-residence	Parental residential status in the family, 3-category (0 = the child living with both parents, 1 = the child living with only one parent, 2 = the child living without parents)
Marital quality of parents	Whether the child’s parents are in good marital relationship (0 = no, 1 = yes)
Family economic condition	Family economic condition reported by parents, 3-category (0 = low income, 1 = medium income, 2 = high income)
Maternal education	Maternal education, 3-category (0 = lower than junior high, 1 = junior high, 2 = higher than junior high)
Paternal education	Paternal education, 3-category (0 = lower than junior high, 1 = junior high, 2 = higher than junior high)
*Hukou* type	The household registration type of the child, dummy variable (0 = rural registration, 1 = urban registration)
Parental age at birth	The age of mother/father at birth of the respondent child, 5 category (0 = 18–24, 1 = 25–29, 2 = 30–34, 3 = 35, and above, 4 = age missing)

#### Key Independent Variables

Our key independent variable is the sibling status. Based on our research objectives, various sibling-related variables were produced. To compare only children with children having siblings, we created a three-category variable named sibship size with only children as the reference group and children having one sibling and children having two or more siblings as the other two groups. We combined the children with two siblings and more into one category (2 + siblings) because there were only five percent of the students having three or more siblings. In addition, to compare only children with children of different birth order from multiple-child families, we created a four-category variable named birth order. Specifically, only children were coded as 0 (reference category); firstborns, middleborns, and lastborns from multiple-child families were coded as 1, 2, and 3, respectively. To be clear, firstborns, middleborns, and lastborns were defined by the birth order of children from multiple-child families: firstborns were children with only younger siblings; middleborns were children who had both younger siblings and older siblings; lastborns were children with only older siblings. See Table 1 for the definitions and measurements of the study variables. At last, to compare only children with children having siblings of different gender, we created another four variables with each having four categories. For example, the variable “younger brothers” indicated whether the child had younger brothers (0 = only child, 1 = without younger brothers, 2 = with younger brothers, 3 = younger brothers missing). The creations of the other three variables (“younger sisters,” “older brothers,” and “older sisters”) followed the same pattern.

#### Potential Moderator

To test whether the effects of only child status on parent–child relationship depend on children’s gender, this study set children’s gender as the moderating variable (0 = son, 1 = daughter).

#### Covariates

We controlled for a variety of covariates in the models. Covariates included adolescents’ demographics (grade and ethnicity), academic characteristics (cognitive score and academic performance), family dynamics (boarding school attendance, parental co-residence, and parental marital quality), family SES (family economic condition, parental education, *hukou* type), and parental age at birth of the respondent child. Children’s grade (grade 7 and grade 9) is a reflection of both children’s age and birth cohort which could influence parent–child closeness as well as sibship size. Children’s academic characteristics were also found to predict parent–child relationship (Sharma and Vaid, 2005), especially in the Chinese culture highly valuing children’s education (Huang and Gove, 2015). According to family systems theory, family structure (boarding school attendance and parental co-residence) and marital relationships were strong predictors of parent–child relationship (Dinisman et al., 2017; Yoo, 2020). Children’s ethnicity and family SES could affect not only parent–child closeness but also sibship size (Zhang, 2012; Piotrowski and Tong, 2016; Weng et al., 2019). The one-child policy were implemented more rigorously in the Han ethnicity than in minority ethnicities and in urban families than in rural families, we therefore included ethnicity and the *hukou* type (Weng et al., 2019). Research has consistently found that with the increase of parental education, the number of children declines (Piotrowski and Tong, 2016) and the parent–child relationship improves (Zhang, 2012). We also controlled for parental age at birth of the surveyed child because it was expected to influence both parent–child relationship and sibship size. Because parental age at birth had a high proportion of missing values (24.61%), we included “age missing” along with other values in the model. Refer to Table 1 for the specific measurements.

### Analytical Strategy

**FIGURE 1:**
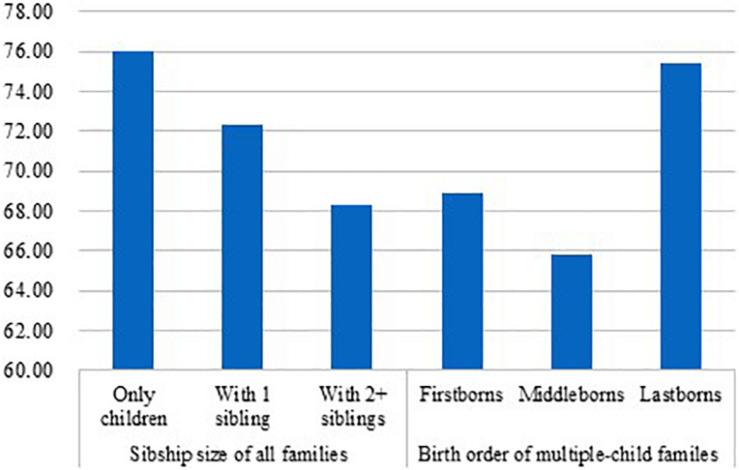
Percent of a “close” mother–child relationship by sibship size and birth order (firstborns do not consist of only children).

**FIGURE 2:**
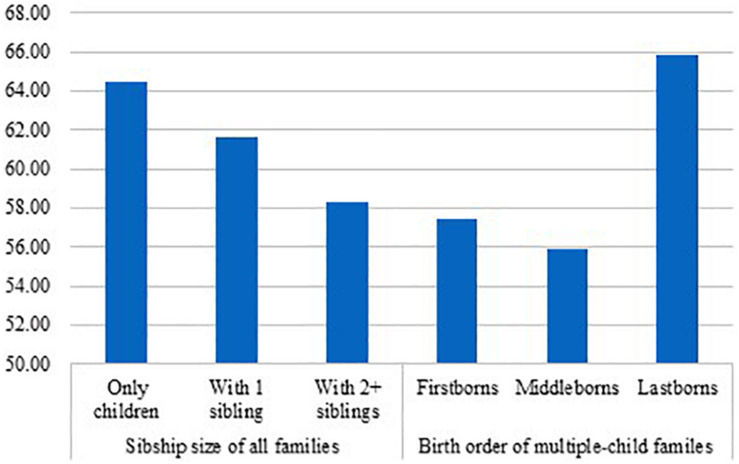
Percent of a “close” father–child relationship by sibship size and birth order (firstborns do not consist of only children).

We started the analyses by reporting the sibling information of the analytical sample (Table 2) and the sample characteristics in the full, only child, and non-only child sample (Table 3). Meanwhile, we displayed the percent of “close” mother–child and father–child relationships by children’s sibship size and birth order (Figures 1, 2). In the next step, given the ordinal nature of the dependent variables, we employed two-level ordered logistic models to estimate mother–child closeness and father–child closeness (Tables 4, 5). Two-level models were used due to the nested structure of the data (students were nested in schools).

**TABLE 2:** Sibling information.

**Variables**	**Percent or Mean/SD**
**Sibship size**	
Only child (ref.)	44.25
Child with 1 sibling	42.00
Child with 2 + siblings	13.76
**Birth order**	
Only child (ref.)	44.25
Firstborn	27.03
Middleborn	5.32
Lastborn	23.40
**Younger brothers**	
Only child (ref.)	44.25
Without younger brother(s)	26.08
With younger brother(s)	21.66
Missing	8.01
**Younger sisters**	
Only child (ref.)	44.25
Without younger sister(s)	31.64
With younger sister(s)	14.13
Missing	9.98
**Older brothers**	
Only child (ref.)	44.25
Without older brother(s)	34.37
With older brother(s)	10.77
Missing	10.62
**Older sisters**	
Only child (ref.)	44.25
Without older sister(s)	27.01
With older sister(s)	20.24
Missing	8.50
Total number of children in the family	1.74/0.82

**TABLE 3:** Sample characteristics (Percent or Mean/SD).

**Variable**	**Full sample (*N* = 18,445)**	**Only children (*N* = 8,161)**	**Non-only children (*N* = 10,284)**
Mother–child relationship*			
Not close (ref.)	2.40	2.21	2.55
Moderate	24.21	21.76	26.15
Close	73.40	76.03	71.30
Father–child relationship*			
Not close (ref.)	4.30	4.30	4.30
Moderate	33.28	31.26	34.88
Close	62.42	64.44	60.82
Child’s gender*			
Son (ref.)	51.34	55.50	48.04
Daughter	48.66	44.50	51.96
**Grade**			
Grade 7 (ref.)	52.55	52.09	52.91
Grade 9	47.45	47.91	47.09
Ethnicity*			
Han (ref.)	91.56	94.82	88.97
Minority	8.44	5.18	11.03
Cognitive score (from −2.03 to 2.71)*	0.02/0.86	0.23/0.85	−0.15/0.83
Academic performance (from 0 to 4)*	2.07/1.12	2.16/1.12	1.99/1.12
Boarding*			
No (ref.)	67.77	84.24	54.70
Yes	32.23	15.76	45.30
Parental co-residence*			
Both parents (ref.)	77.40	82.51	73.34
One parent	12.36	12.14	12.53
No parent	10.24	5.34	14.13
Marital quality of parents*			
Not good (ref.)	16.40	17.18	15.78
Good	83.60	82.82	84.22
Family economic condition*			
Low income (ref.)	20.46	11.74	27.37
Medium income	73.46	80.84	67.61
High income	6.08	7.43	5.02
Maternal education*			
<Junior high (ref.)	24.40	10.48	35.45
=Junior high	40.88	35.11	45.46
> Junior high	34.72	54.42	19.09
Paternal education*			
<Junior high (ref.)	15.00	7.99	20.56
=Junior high	44.08	32.36	53.38
>Junior high	40.92	59.65	26.06
*Hukou* type*			
Rural (ref.)	54.46	33.27	71.28
Urban	45.54	66.73	28.72
Parental age at birth*			
18–24	23.93	25.99	22.29
25–29	35.19	42.87	29.08
30–34	12.29	10.40	13.78
≥35	4.00	2.59	5.11
Missing	24.61	18.15	29.74

*In the first column signals a significant difference between only and non-only children (*p* < 0.05). Significance of difference for each variable is determined by chi-squared test (for categorical variables) or *t*-test (for continuous variables).

**TABLE 4:** Two-level ordered logistic models estimating mother–child closeness (*N* = 18445).

	**Sibship size**			**Birth order**		
	**Model a1**	**Model a2**	**Model a3**	**Model b1**	**Model b2**	**Model b3**
***Fixed effects***						
Child with 1 sibling (only child)	−0.12**	−0.11*	0.03			
	(0.04)	(0.04)	(0.06)			
Child with 2 + siblings (only child)	−0.25***	−0.18**	0.14			
	(0.06)	(0.06)	(0.08)			
Firstborn (only child)				−0.21**	−0.29***	–0.15
				(0.07)	(0.07)	(0.08)
Middleborn (only child)				–0.24	−0.27*	–0.08
				(0.13)	(0.13)	(0.17)
Lastborn (only child)				0.11	0.15	0.28***
				(0.07)	(0.07)	(0.08)
Total number of children in the family				–0.05	–0.00	–0.00
				(0.04)	(0.04)	(0.04)
Daughter		0.11**	0.36***		0.17***	0.37***
		(0.04)	(0.06)		(0.04)	(0.06)
Grade 9		−0.34***	−0.33***		−0.34***	−0.33***
		(0.04)	(0.04)		(0.04)	(0.04)
Minority		–0.07	–0.07		–0.06	–0.06
		(0.08)	(0.08)		(0.08)	(0.08)
Cognitive score		–0.01	–0.01		–0.01	–0.01
		(0.02)	(0.02)		(0.02)	(0.02)
Academic performance		0.18***	0.19***		0.19***	0.19***
		(0.02)	(0.02)		(0.02)	(0.02)
Boarding		0.28***	0.29***		0.29***	0.29***
		(0.06)	(0.06)		(0.06)	(0.06)
Living with one parent (both parents)		−0.25***	−0.26***		−0.25***	−0.26***
		(0.05)	(0.05)		(0.05)	(0.05)
Living with no parent (both parents)		−0.43***	−0.43***		−0.42***	−0.42***
		(0.06)	(0.06)		(0.06)	(0.06)
Good marital quality of parents		0.98***	0.98***		0.98***	0.98***
		(0.04)	(0.04)		(0.04)	(0.04)
Medium income (low)		0.14**	0.14**		0.13**	0.13**
		(0.05)	(0.05)		(0.05)	(0.05)
High income (low)		0.17	0.16		0.16	0.15
		(0.09)	(0.09)		(0.09)	(0.09)
Maternal education is junior high (<Junior high)		0.20***	0.20***		0.21***	0.21***
		(0.05)	(0.05)		(0.05)	(0.05)
Maternal education higher than junior high (<Junior high)		0.27***	0.26***		0.29***	0.28***
		(0.06)	(0.06)		(0.06)	(0.06)
Paternal education is junior high (<Junior high)		0.04	0.04		0.06	0.06
		(0.05)	(0.05)		(0.05)	(0.05)
Paternal education higher than junior high (<Junior high)		0.13*	0.13*		0.15*	0.15*
		(0.06)	(0.06)		(0.06)	(0.06)
Urban *hukou*		–0.00	–0.01		–0.00	–0.01
		(0.04)	(0.04)		(0.04)	(0.04)
Parental age at birth was 25–29 (18–24)		–0.04	–0.05		−0.11*	−0.11*
		(0.05)	(0.05)		(0.05)	(0.05)
Parental age at birth was 30–34 (18–24)		0.03	0.01		–0.13	–0.13
		(0.06)	(0.06)		(0.07)	(0.07)
***Fixed effects***						
Parental age at birth was 35 and above (18–24)		0.10	0.07		–0.11	–0.11
		(0.10)	(0.10)		(0.10)	(0.10)
Parental age at birth was missing (18–24)		−0.20***	−0.22***		−0.27***	−0.27***
		(0.05)	(0.05)		(0.05)	(0.05)
**Interactions**						
Child with 1 sibling × Daughter			−0.34***			
			(0.08)			
Child with 2 + siblings × Daughter			−0.64***			
			(0.11)			
Firstborn × Daughter						−0.31***
						(0.09)
Middleborn × Daughter						−0.37*
						(0.16)
Lastborn × Daughter						−0.37***
						(0.09)
***Random effects***						
Between-school variance	0.21***	0.17***	0.17***	0.21***	0.16***	0.17***
	(0.03)	(0.03)	(0.03)	(0.03)	(0.03)	(0.03)
Observations	18,445	18,445	18,445	18,445	18,445	18,445
Number of groups	112	112	112	112	112	112

Reference categories in the parentheses of the first column; Standard errors in parentheses.

***p < 0.001, **p < 0.01, *p < 0.05.

**TABLE 5:** Two-level ordered logistic models estimating father–child closeness (*N* = 18,445).

	**Sibship size**			**Birth order**		
	**Model a1**	**Model a2**	**Model a3**	**Model b1**	**Model b2**	**Model b3**
***Fixed effects***						
Child with 1 sibling (only child)	−0.12**	−0.15***	–0.03			
	(0.04)	(0.04)	(0.05)			
Child with 2 + siblings (only child)	−0.20***	−0.18**	0.15			
	(0.05)	(0.06)	(0.08)			
Firstborn (only child)				−0.24***	−0.29***	−0.16*
				(0.06)	(0.06)	(0.07)
Middleborn (only child)				–0.19	–0.22	0.08
				(0.12)	(0.12)	(0.16)
Lastborn (only child)				0.11	0.05	0.17*
				(0.06)	(0.07)	(0.08)
Total Number of children in the family				–0.03	–0.00	–0.01
				(0.04)	(0.04)	(0.04)
Daughter		−0.13***	0.07		−0.09**	0.07
		(0.03)	(0.05)		(0.03)	(0.05)
Grade 9		−0.33***	−0.33***		−0.33***	−0.33***
		(0.03)	(0.03)		(0.03)	(0.03)
Minority		0.08	0.07		0.08	0.07
		(0.08)	(0.08)		(0.08)	(0.08)
Cognitive score		–0.03	–0.04		–0.03	–0.04
		(0.02)	(0.02)		(0.02)	(0.02)
Academic performance		0.12***	0.12***		0.12***	0.12***
		(0.02)	(0.02)		(0.02)	(0.02)
Boarding		0.17**	0.18***		0.17***	0.18***
		(0.05)	(0.05)		(0.05)	(0.05)
Living with one parent (both parents)		−0.53***	−0.54***		−0.54***	−0.54***
		(0.05)	(0.05)		(0.05)	(0.05)
Living with no parent (both parents)		−0.29***	−0.29***		−0.28***	−0.28***
		(0.06)	(0.06)		(0.06)	(0.06)
Good marital quality of parents		1.11***	1.11***		1.11***	1.11***
		(0.04)	(0.04)		(0.04)	(0.04)
Medium income (low)		0.05	0.05		0.04	0.04
		(0.04)	(0.04)		(0.04)	(0.04)
High income (low)		0.13	0.13		0.12	0.12
		(0.08)	(0.08)		(0.08)	(0.08)
Maternal education is junior high (<Junior high)		0.09*	0.09*		0.10*	0.10*
		(0.04)	(0.04)		(0.04)	(0.04)
Maternal education higher than junior high (<Junior high)		0.15**	0.15**		0.17**	0.16**
		(0.06)	(0.06)		(0.06)	(0.06)
Paternal education is junior high (<Junior high)		0.18***	0.18***		0.19***	0.19***
		(0.05)	(0.05)		(0.05)	(0.05)
Paternal education higher than junior high (<Junior high)		0.25***	0.24***		0.26***	0.26***
		(0.06)	(0.06)		(0.06)	(0.06)
Urban *hukou*		–0.07	−0.08*		–0.07	–0.08
		(0.04)	(0.04)		(0.04)	(0.04)
Parental age at birth was 25–29 (18–24)		0.03	0.02		–0.02	–0.02
		(0.04)	(0.04)		(0.04)	(0.04)
Parental age at birth was 30–34 (18–24)		0.21***	0.19**		0.08	0.08
		(0.06)	(0.06)		(0.06)	(0.06)
***Fixed effects***						
Parental age at birth was 35 and above (18–24)		0.36***	0.34***		0.20*	0.21*
		(0.09)	(0.09)		(0.09)	(0.09)
Parental age at birth was missing (18–24)		–0.02	–0.03		–0.07	–0.07
		(0.05)	(0.05)		(0.05)	(0.05)
**Interactions**						
Child with 1 sibling × Daughter			−0.26***			
			(0.07)			
Child with 2 + siblings × Daughter			−0.61***			
			(0.10)			
Firstborn × Daughter						−0.25**
						(0.08)
Middleborn × Daughter						−0.48**
						(0.15)
Lastborn × Daughter						−0.30***
						(0.08)
***Random effects***						
Between-school variance	0.15***	0.12***	0.12***	0.15***	0.12***	0.12***
	(0.02)	(0.02)	(0.02)	(0.02)	(0.02)	(0.02)
Observations	18,445	18,445	18,445	18,445	18,445	18,445
Number of groups	112	112	112	112	112	112

Reference categories in the parentheses of the first column; Standard errors in parentheses.

***p < 0.001, **p < 0.01, *p < 0.05.

## Results

### Descriptive Analyses

Table 2 reports the sibling information of our analytical sample. Information in Table 2 indicates that modern Chinese families have a very small family size with one-child and two-child families accounting for a large proportion (more than 80%). Specifically, only children accounted for almost half of the sampled children (44.25%); children with only one sibling accounted for 42% of the full sample; children with two or more siblings held a very low proportion of 14%. Of the analytical children, around 27% were firstborns, 5% were middleborns, and 23% were lastborns. Among our sampled children, those having younger brothers held the largest proportion (21.66%) and those having older sisters accounted for the second largest proportion (20.24%). Only 10.77% of the children had older brothers. This is perhaps because most rural parents were subject to the one-and-a-half-child policy: rural couples whose first child was a daughter were allowed to have a second child, whereas those with a son as the first child were not allowed to have another child (Jiang and Liu, 2016). The mean number of children for each household in our sample was only 1.74.

Table 3 reports the sample characteristics. In addition to showing the sample characteristics in the full sample, Table 3 also displays the characteristics by children’s only child status. Meanwhile, the chi-squared test (for categorical variables) or *t*-test (for continuous variables) was employed to decide if the difference between only children and non-only children were significant. As shown in Table 3, most junior-high-school students had a close parent–child relationship (73.40% for mother–child relationship and 62.42% for father–child relationship). Chi-squared tests show that only children were significantly different from non-only children in three levels of mother–child relationship (χ^2^ = 52.23, df = 2, *p* = 0.000) and father–child relationship (χ^2^ = 27.47, df = 2, *p* = 0.000). To test whether only children were significantly different from non-only children in reporting “close” parent–child relationships, we combined “not close” and “moderate” into one category. After the combination, chi-squared tests of the two levels of parent–child relationships (“not close-moderate combination” and “close”) show that compared to non-only children (71.30%), only children (76.03%) were more likely to report “close” relationships with their mothers (χ^2^ = 52.08, df = 1, *p* = 0.000); compared to non-only children (60.82%), only children (64.44%) were also more likely to report “close” relationships with their fathers (χ^2^ = 25.39, df = 1, *p* = 0.000). In addition, only children had significantly higher cognitive score (0.23 for only children and −0.15 for non-only children, *t* = 30.90, df = 18,443, *p* = 0.000) and reported better academic performance (2.16 for only children and 1.99 for non-only children, *t* = 10.25, df = 18,443, *p* = 0.000) than did non-only children. Non-only children were more likely to attend a boarding school than only children (χ^2^ = 1,800, df = 1, *p* = 0.000). Regarding family background, only children were more likely to be born in high-income families (χ^2^ = 46.19, df = 1, *p* = 0.000), having parents of more educated (maternal education: χ^2^ = 2,500, df = 1, *p* = 0.000; paternal education: χ^2^ = 2,100, df = 1, *p* = 0.000), and having higher probability of living with both parents (χ^2^ = 219.06, df = 1, *p* = 0.000). Finally, due to the more rigorous implementation of the OCP and the more modern culture in urban areas than in rural areas, only children were significantly different from non-only children in hukou type (urban *hukou* accounted for 66.73% among only children and only 28.72% among non-only children, χ^2^ = 2,700, df = 1, *p* = 0.000). Overall, only children were more advantaged in terms of both parent–child relationship and background characteristics than non-only children.

Figures 1, 2 show the percent of “close” mother–child relationship and “close” father–child relationship, respectively, by sibship size and birth order. For “close” mother–child relationship (Figure 1), significant difference was not only found between only children and children with two or more siblings (only children: 76.03%, children with two or more siblings: 68.28%, χ^2^ = 60.73, df = 1, *p* = 0.000) but also found between only children and children having only one sibling (only children: 76.03%, children with 1 sibling: 72.30%; χ^2^ = 29.00, df = 1, *p* = 0.000). Further, only children were also significantly more likely to report “close” mother–child relationships than firstborns (firstborns: 68.87%, χ^2^ = 81.10, df = 1, *p* = 0.000) and middleborns (middleborns: 65.78%, χ^2^ = 49.01, df = 1, *p* = 0.000), but no significant difference was observed between only children and lastborns (lastborns: 75.37%, χ^2^ = 0.67, df = 1, *p* = 0.412). For “close” father–child relationship (Figure 2), the pattern was similar. First, only children were significantly more likely to report “close” father–child relationship than children with one sibling (only children: 64.44%, children with 1 sibling: 61.66%; χ^2^ = 13.22, df = 1, *p* = 0.000) and children with two or more siblings (only children: 64.44%, children with two or more siblings: 58.27%; χ^2^ = 31.57, df = 1, *p* = 0.000). Turing to birth order, only children were also more likely to report “close” father–child relationship than firstborns (firstborns: 57.46%, χ^2^ = 63.87, df = 1, *p* = 0.000) and middleborns (middleborns: 55.91%, χ^2^ = 27.55, df = 1, *p* = 0.000). However, no significant difference was detected between only children and lastborns (lastborns: 65.82%, χ^2^ = 2.38, df = 1, *p* = 0.123).

### Multivariate Analyses

#### Mother–Child Closeness

Table 4 shows the coefficients of two-level ordered logistic models estimating mother–child closeness. Model a1 and Model b1 were designed to test the effects of children’s sibship size and birth order on mother–child closeness without controlling for covariates, respectively. Sibship size and birth order were not included in the models simultaneously in order to avoid multi-collinearity because the two variables shared a same reference group (only children). Model a2 and Model b2 were models estimating the net effects of sibship size and birth order, respectively, with other things being equal (all the covariates were controlled). It is worth noting that, in the birth order model (Model b2), we controlled for the total number of children in the family to capture the net effects of birth order. Model a3 and Model b3 were interaction models designed to test the moderating effects of children’s gender on the effects of sibship size and birth order, respectively.

**FIGURE 3:**
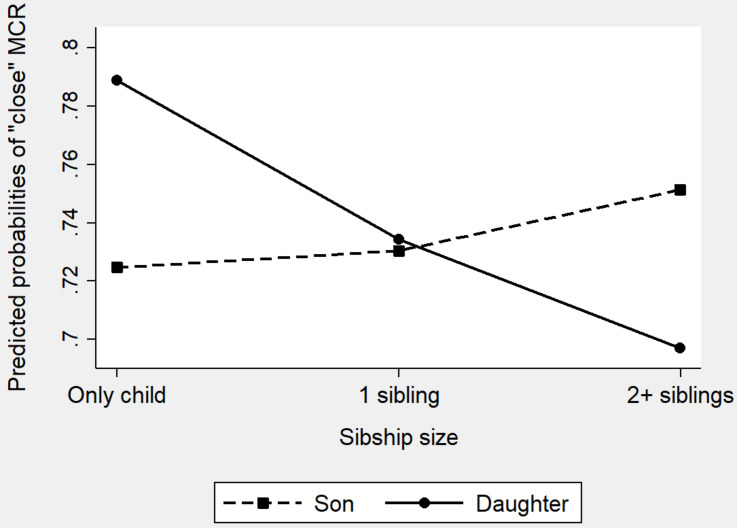
Predicted probabilities of a “close” mother–child relationship by sibship size and children’s gender (MCR: mother-child relationship).

In Model a1, the significantly negative coefficients of one-sibling child and two-or-more-sibling child indicate that the presence of sibling(s) was disadvantaged for children. We then successively added the control variables. In Model a2, with all the covariates being controlled, the negative effects of sibship size dropped in the magnitude but still remained significant. We found that the sibship size effects were largely confounded by family SES (results not shown). Other things being equal, compared to only children, children with one sibling were 10% [1- exp (−0.11), *p* = 0.016] less likely to report a close relationship with their mothers; Children with two or more siblings were 16.5% [1- exp (−0.18), *p* = 0.004] less likely to report a close mother–child relationship. In addition, the significantly positive coefficient of children’s gender (β = 0.11, *p* = 0.001) implied that daughters were more likely to report a close mother–child relationship than sons. Moving to Model a3, the coefficients of the interaction terms are significantly negative (β_1__sibling__×__daughter_ = −0.34, *p* = 0.000; β_2__+ siblings__×__daughter_ = −0.64, *p* = 0.000) indicating that the effects of sibship size were significantly different between daughters and sons. We visually displayed the interaction effects in the form of predicted probabilities (for “close” mother–child closeness) in Figure 3. Figure 3 clearly shows that, the changing directions of the solid line (representing daughter) and the dash line (representing son) were different. Larger sibship size reduced daughters’ probabilities of having a close relationship with mothers by a great degree whereas slightly increased sons’ probabilities of attaining such relationship. In other words, the benefits of being an only child is mainly reflected on daughters in the Chinese context.

**FIGURE 4:**
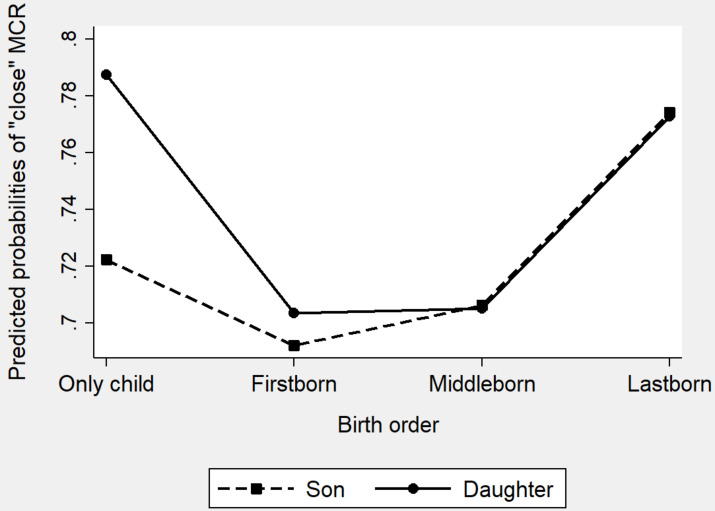
Predicted probabilities of a “close” mother–child relationship by birth order and children’s gender (MCR: mother-child relationship).

Turning to the birth-order models. In Model b1, without controlling for other variables, firstborns were found to be less likely to form a close mother–child relationship compared to only children. In Model b2, net of all the other factors, compared to only children, firstborns and middleborns were 25% [1- exp (−0.29), *p* = 0.000] and 24% [1- exp (−0.27), *p* = 0.041] less likely, respectively, to have a close mother–child relationship. Finally, the coefficient of lastborns is positive and marginally significant (β = 0.15; *p* = 0.052) suggesting lastborns were not disadvantaged compared to only children in mother–child closeness. Turning to Model b3 with interaction terms, we found a significant joint effects of birth order with children’s gender. Figure 4 clearly shows the interaction information of Model b5: daughters as only children had a significantly higher probability to enjoy a close mother–child relationship than sons as only children. Last daughters and sons had the same probability to enjoy a close mother–child relationship. Firstborns and middleborns (both daughters and sons) were least likely to have a close mother–child relationship.

#### Father–Child Closeness

Table 5 shows the coefficients of two-level logistic regression estimating father–child closeness. Model a1 and Model b1 were designed to test the sibship-size effects and birth-order effects on father–child closeness without controlling for other variables, respectively. Model a2 and Model b2 were models testing the net effects of sibship size and birth order (all covariates were controlled). Similar to the estimates of mother–child relationship, sibship size and birth order were not included simultaneously to avoid multi-collinearity. Model a3 and Model b3 were interaction models testing whether children’s gender moderated the sibship-size effects and birth-order effects, respectively.

**FIGURE 5:**
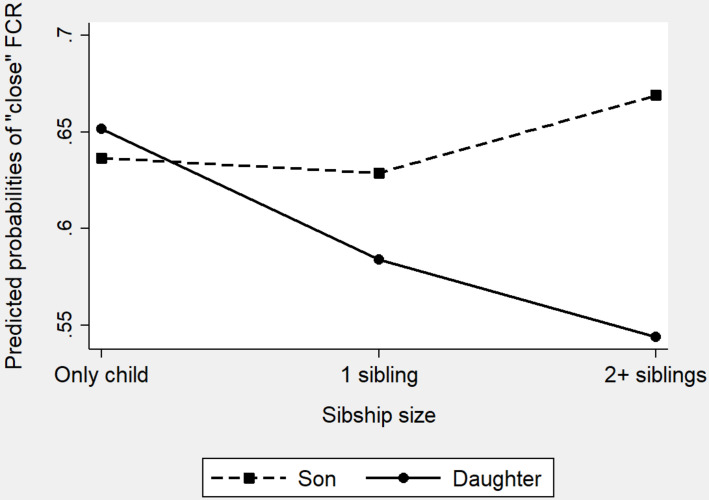
Predicted probabilities of a “close” father–child relationship by sibship size and children’s gender (FCR: father-child relationship).

In Model a1, the coefficients of sibship size were significantly negative suggesting that compared to only children, children with siblings experienced a declined odds of having a close father–child relationship. We then successively added covariates in the model with Model a2 including all variables. Holding other things consistent, having one sibling and two or more siblings reduced the odds of enjoying a close father–child relationship by 14% [1-exp (−0.15), *p* = 0.000] and 16% [1-exp (−0.18), *p* = 0.002], respectively. It is worth noting the coefficient of children’s gender: although daughters were more likely (β = 0.11, *p* = 0.001) to have a close mother–child relationship than sons (see Model a2 in Table 4), they were less likely (β = −0.13, *p* = 0.000) to have a close father–child relationship. Turning to Model a3, the significant coefficients of the interaction terms suggest that children’s gender and sibship size jointly influenced father–child relationship (β_1__sibling__×__daughter_ = −0.26, *p* = 0.000; β_2__+ siblings__×__daughter_ = −0.61, *p* = 0.000). We visually displayed the interaction information of Model a3 in Figure 5. Figure 5 clearly shows that daughters’ probabilities of having a “close” father–child relation declined with the increase of sibship size and only daughters have the highest probabilities. Sons, on the contrary, experienced a slightly increase in father-son closeness as their sibship size rose. Among non-only children (children with 1 sibling or 2+ siblings), sons had higher probabilities of reporting a close father–child relationship than did daughters, whereas among children without siblings, daughters had higher probabilities in reporting a close relationship with their fathers than did sons. Figure 5 suggests that daughters, rather than sons, benefit from being only children.

**FIGURE 6:**
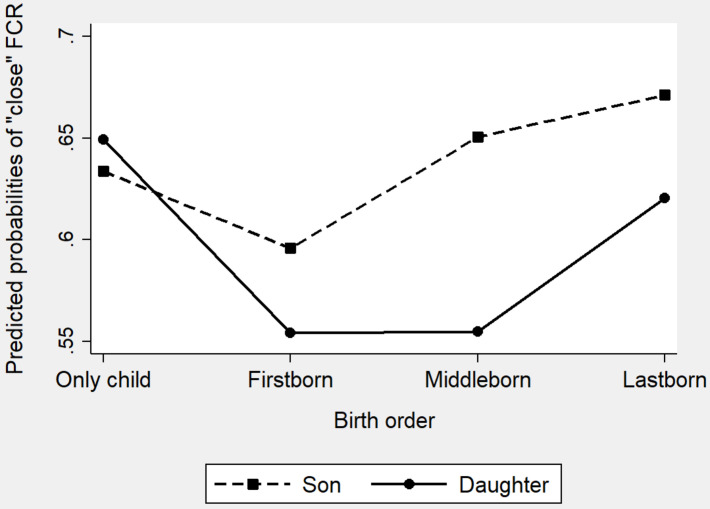
Predicted probabilities of a “close” father–child relationship by birth order and children’s gender (FCR: father-child relationship).

Model b1 (only including birth order) suggests that only children were significantly more likely to have close father–child relationships than did firstborns. In Model b2, with all the covariates being controlled, firstborns were 25% [1-exp (−0.29), *p* = 0.000] less likely to report a close father–child relationship. However, there was no significant difference between only children and middleborns or lastborns. Model b3 includes the interactions of children’s gender and birth order to test whether birth order influenced father–child relationships differently for daughters and sons. The coefficients of the interactions were significantly negative suggesting daughters and sons showed different patterns in the association between birth order and father–child relationship. We displayed the interaction information of Model 4 in Figure 6. Figure 6 clearly shows that, for sons, being the lastborns of multiple-child families was most beneficial. This is probably due to the son preference: the youngest sons in the families were usually born in the situation that fathers were dissatisfied with the number of sons and their births would make up for it (Basu and De Jong, 2010). Therefore, the births of younger sons would bring about more satisfactions than that of older sons. However, for daughters, the situation is distinct: being the only child was most beneficial. This is also an indirect reflection of son preference: only when there were no siblings to compete for family resources will daughters receive more attention from parents in the Chinese families.

#### The Presence of Siblings of Different Gender

To compare only children with children having siblings of different gender, we ran a series of additional models. See Table 6.

**TABLE 6:** Effects of younger brothers, younger sisters, older brothers, and older sisters.

**Variables**	**Mother–child closeness**		**Father–child closeness**	
	**Main effects models**	**Interaction effects models**	**Main effects models**	**Interaction effects models**
**Models testing the effects of younger brothers**				
Only children (ref.)				
Without younger brother(s)	0.08	0.21**	–0.06	0.06
With younger brother(s)	−0.40***	−0.25**	−0.34***	−0.18*
Missing	–0.15	–0.04	–0.14	0
Younger brother(s) × Daughter				
Without × Daughter		−0.35***		−0.28***
With × Daughter		−0.30***		−0.29***
Missing × Daughter		−0.27*		−0.32**
**Models testing the effects of younger sisters**				
Only children (ref.)				
Without younger sister(s)	–0.02	0.17*	–0.08	0.07
With younger sister(s)	−0.19*	–0.06	−0.29***	–0.15
Missing	−0.18*	0	−0.23***	–0.04
Younger sister(s) × Daughter				
Without × Daughter		−0.46***		−0.34***
With × Daughter		−0.33**		−0.29**
Missing × Daughter		−0.41***		−0.39***
**Models testing the effects of older brothers**				
Only children (ref.)				
Without older brother(s)	–0.04	0.14	−0.11*	0.03
With older brother(s)	0.06	0.21*	0.02	0.22*
Missing	–0.20	–0.05	−0.23**	–0.09
Older brother(s) × Daughter				
Without × Daughter		−0.45***		−0.33***
With × Daughter		−0.37**		−0.42***
Missing × Daughter		−0.36**		−0.31**
**Models testing the effects of older sisters**				
Only children (ref.)				
Without older sister(s)	–0.08	0.05	−0.12*	0
With older sister(s)	0.29***	0.42***	0.10	0.22**
Missing	−0.16*	–0.05	−0.24**	–0.12
Older sister(s) × Daughter				
Without × Daughter		−0.33***		−0.28***
With × Daughter		−0.41***		−0.35***
Missing × Daughter		−0.28*		−0.27*

All control variables are included in the models (control variables are the same as those included in the birth-order models), but now shown.

***p < 0.001, **p < 0.01, *p < 0.05.

The models shown in the left part of Table 6 estimate mother–child closeness. In the main-effects models, having younger brothers and having younger sisters reduced the odds of involving in a close mother–child relationship by 33% [1-exp (−0.40), *p* = 0.000] and 17% [1-exp (−0.19), *p* = 0.015], respectively, whereas having older sisters were a favorable factor [increasing the odds by 34%, exp (0.29)-1, *p* = 0.000]. Moving to the interaction models of younger brother(s) and younger sister(s), the coefficients of the interaction terms “with younger brother(s) × daughter” and “with younger sister(s) × daughter” were both significantly negative [β_with younger  brother(s)__×__daughter_ = −0.30, *p* = 0.001; β_with  younger  sister(s)__×__daughter_ = −0.33, *p* = 0.002] suggesting having younger siblings exerted stronger negative impacts on daughters than on sons. In the interaction model of older brother(s), the main effects of older brother(s) was significantly positive [β_with  older  brother(s)_ = 0.21, *p* = 0.044] suggesting that for sons, older brother(s) presence was an advantageous factor in mother–child closeness, whereas the interaction effects was significantly negative [β_with  older  brother(s)__×__daughter_ = −0.37, *p* = 0.002] suggesting having older brothers was an unfavorable factor for daughters. Coefficients of the interaction term in the interaction model of older sister(s) suggested that the positive effect of having older sisters was stronger for sons than for daughters [β_with  older  sister(s)_ = 0.42, *p* = 0.000; β_with  older  sister(s)__×__daughter_ = −0.41, *p* = 0.000].

The models shown in the right part of Table 6 predict father–child closeness. The main-effects analyses revealed that having younger siblings was an unfavorable factor for children to have a close father–child relationship, compared to only children [β_with  younger  brother(s)_ = −0.34, *p* = 0.000; β_with  younger  sister(s)_ = −0.29, *p* = 0.000]. Meanwhile, the effects of having older siblings were insignificant. Turning to the interaction models. Interaction analyses of younger siblings suggested that the negative effects of having younger siblings were stronger for daughters than for sons [β_with  younger  brother(s)__×__daughter_ = −0.29, *p* = 0.001; β_with  younger  sister(s)__×__daughter_ = −0.29, *p* = 0.002]. Interaction analyses of older siblings suggested that although having older siblings did not matter for the overall children, interesting patterns emerged when we included interaction terms: the presence of older siblings was positively associated with sons’ closeness to fathers [β_with  older  brother(s)_ = 0.22, *p* = 0.024; β_with  older  sister(s)_ = 0.22, *p* = 0.004], whereas negatively linked to daughters’ closeness to fathers [β_with  older  brother(s)__×__daughter_ = −0.42, *p* = 0.000; β_with  older  sister(s)__×__daughter_ = −0.35, *p* = 0.000].

In summary, influenced by son preference, the associations between the presence of siblings and parent–child closeness were based on different sibling-gender compositions. Specifically, having younger brothers was an unfavorable factor for children regarding parent-child relationship, especially for female children. By comparison, having older sisters was a favorable factor for male children in terms of parent-child relationship.

## Discussion

After more than three decades of implementation, the one-child policy created a large number of families with just one child and this family size became the social norm in Chinese society (Falbo and Hooper, 2015; Falbo, 2018). A growing body of research has documented the developmental outcomes of being an only child (Liu et al., 2010; Falbo, 2012; Falbo and Hooper, 2015; Guo et al., 2015, 2018; Wang et al., 2020). However, few studies examined whether only children have different parent–child emotional relationships compared with non-only children. As an emotional resource, the parent–child relationship plays an important role in shaping adolescents’ psychological, social, and academic development (Li et al., 2018; Xu et al., 2019). Thus, it is essential to gain insight into only children’s relationships with their parents. Western research has reported that although only children are generally more advantaged in the parent–child relationship compared to non-only children, only children are no different from firstborns or those from two-child families (Falbo and Polit, 1986). Therefore, in addition to exploring the sibship-size effects on parent–child relationships, the birth-order effects should also be tested in the Chinese context. Considering the gender stratification in Chinese culture (Guo et al., 2018), this study further examined whether the sibship-size effects and birth-order effects depended on children’s gender. Finally, comparisons between only children and children with siblings of different gender were also made. The findings of this study are as follows.

First, Chinese only children had closer parent–child relationship than did non-only children. Specifically, compared to children from two-child families or larger families, only children were more likely to describe their relationships with their mothers and fathers as highly close. This result is consistent with the resource dilution theory (Blake, 1981). The quality of the parent–child relationship tends to be a reflection of parental time, energy, and attention devoted to children and such resources are not infinite (Li et al., 2015). Therefore, children with many siblings have to share these resources with their siblings and, in this case, the resources for each child would decrease; on the contrary, only children do not need to compete with their siblings and thus have more access to family resources (Downey, 1995). As a result, only children are more likely to describe their relationships with their parents as close, suggesting that the parent–child relationships for only children are of higher quality than those found among other sibship sizes. This result demonstrates the existence of the child-centered culture in Chinese one-child families (Tsui and Rich, 2002). Previous studies have found that only children have higher educational achievements than non-only children because the former receive more educational resources from parents (Downey, 1995; Lee, 2012). This study contributes to the existing literature by including parental emotional resources in the resource-dilution model.

Second, considering birth order, we found that only children had advantages over firstborns (of multiple-child families), but these advantages disappeared when they were compared to lastborns (of multiple-child families). This is inconsistent with findings from Western families (Falbo and Polit, 1986). In the West, only children were found to be indistinguishable from firstborns in terms of parent–child relationships because, before the births of younger siblings, firstborns are the only children of their parents, at least for a while, and have access to all the family resources at that period (Falbo and Polit, 1986; Mellor, 1990; Falbo, 2012). Therefore, in Western families, only children are more advantaged only when they are compared to laterborns of large families with three or more children (Falbo, 2012). However, our study demonstrates that the situation is different in Chinese families: Chinese only children are more at an advantage than firstborns and they are no different from lastborns. In short, among all children, firstborns as well as middleborns of multiple-child families are the most disadvantaged. The following are some possible explanations for this result.

The first explanation concerns Chinese culture. The traditional Confucian idea that “having many children is a blessing” is embraced by many Chinese parents, especially those from rural areas (Hillier, 1988; Jiao et al., 1996). In this case, parents tend to violate the One-Child Policy to have an ideal composition of gender and the number of children (Jiao et al., 1996). As a consequence, the last child is usually the one parents show more affection toward. Another explanation is related to the personality traits of children. According to Sulloway’s (1996) theory about birth order, children of different order usually have different personalities. Firstborns, being the oldest in the family, are expected to act as a leader (Shao et al., 2013). This is particularly true in China: historically, the eldest son is second in authority to his father in Chinese families (Das Gupta et al., 2003). This requires the eldest children to be responsible, independent, and conservative (Shao et al., 2013). The youngest children, on the other hand, were found to be higher in sociability (Sulloway, 1996; Shao et al., 2013). Therefore, lastborns tend to be more open than firstborns– this argument has been demonstrated in the Chinese context (Shao et al., 2013). Being more open enables lastborns to be more likely attaining parental attention, which results in a higher parent–child closeness.

Third, the parent–child relationship was significantly associated with children’s gender and such association differed by parental gender: daughters had a significantly more positive relationship with their mothers than sons, whereas sons had a significantly more positive relationship with their fathers than daughters. In short, parents were emotionally closer to their same-sex children. This pattern is consistent with the gendered theory of parenting (Liu, 2020). According to the gendered theory of parenting, the gender of children and parents may simultaneously influence parenting styles as well as parent–child relationships (Russell and Saebel, 1997). This gendered pattern may be reinforced in the Chinese culture characterized by traditional gender stereotypes or gender-specific expectations: the boys should be manliness and hard and the girls should be gentle and soft (Liu, 2006). Encouraged by this notion, fathers usually spend more time with their sons to cultivate their masculinity and mothers tend to spend more time with their daughters to nurture their femininity (Liu, 2006). Therefore, the “same-sex parent–child dyads” phenomenon is popular in Chinese families.

Forth, the only child status influenced the parent–child relationship depending on children’s gender. Specifically, even though having siblings was an obstructive factor for children in general to get a closer parent–child relationship, this negative effect was stronger for daughters than for sons. In short, daughters were more responsive to sibling status and benefited more from being only children. This could again be explained by the patriarchal culture derived from Confucianism (Das Gupta et al., 2003). According to Patriarchy, Chinese families value sons’ roles as providers of old-age-support for their parents while devaluing daughters’ roles because their supports would eventually be channeled to their husband’s families (Deutsch, 2006; Xie, 2013). In this case, parents would invest more in sons than in daughters to maximize the benefits of investment (Jiang et al., 2012). Therefore, the more siblings the daughters have, the fewer resources they would receive (Chu et al., 2007). Research has found that the resource dilution model is characterized by a gendered pattern in Chinese families: the negative effects of sibling presence is stronger for daughters than for sons (Chu et al., 2007; Lee, 2012). Nevertheless, when the family has only one child, the gender of the child does not matter for the parenting strategies (Tsui and Rich, 2002). This is perhaps because parents of only daughters usually hold more egalitarian gender role attitudes given that they had stopped giving birth in the case of having no sons. Moreover, recent studies have found that it is increasingly becoming common for daughters to transfer money or provide care to their aging parents in both rural and urban China (Xie and Zhu, 2009; Gruijters, 2018). Thus, modern parents have economic incentives to invest in their only daughters (Tsui and Rich, 2002). The gender bias weakens in one-child families (Fong, 2002). Furthermore, considering the gender discrimination in the labor market, women may need more skills to compete with men (Raley and Bianchi, 2006). Therefore, to guarantee the future success of their daughters, parents of singleton girls may have higher incentives to invest in their children than parents of singleton boys (Tsui and Rich, 2002). Thus, the advantages of singleton daughters could be considered a proactive strategy to prepare for discrimination against women in the job market.

At last, our additional analyses found that the sibling effects on parent–child closeness differed by sibling-gender composition: female children were more likely to be disadvantaged due to the presence of younger brothers, whereas male children could benefit more from having older sisters. Previous studies, based on the son preference culture, has developed the resource dilution theory in China by introducing gender of siblings (Chu et al., 2007; Zheng, 2015). These studies found that siblings were not equally associated with one’s educational resources: brothers reduced educational opportunities, while sisters increased one’s educational opportunities (Chu et al., 2007; Zheng, 2015). Therefore, the resource dilution is gender asymmetric in the Chinese culture (Zheng, 2015). Our study has developed the theory by examining the parent–child relationship: besides educational opportunities, the gender asymmetric pattern was also found for parent–child closeness. Under the son preference culture of Asian countries, parents, especially rural parents, would stop giving birth only when the desired number of sons was achieved (“male-preferring stopping rules”) (Basu and De Jong, 2010). In this situation, families with only daughters were usually unsatisfied with the gender composition and would continue to give birth in their unrelenting search for a son—leading daughters usually being born at earlier parities within families (Basu and De Jong, 2010). This could also be reflected in our data that children having younger brothers or older sisters accounted for the largest proportions, whereas children having older brothers were the least. This idea was again reinforced by Chinese national policy (the one-and-a-half-child policy which allowed rural couples to have a second child if the first child was a girl, see Jiang and Liu, 2016). As a result, parents are more likely to value their youngest male children who have older sisters and devalue their eldest female children who have younger brothers. Our findings suggest that despite daughters’ status has been improved in one-child families, son preference and daughter discrimination still persist in multiple-child families in modern China.

### Limitation

As with any study, the current study has some limitations. First, due to the cross-sectional nature of the dataset, one should be very cautious to conclude a causal relationship between only child status and parent–child relationship. Both children’s only child status and parental relationship with children are determined by parental characteristics that were not fully captured by our data. Second, due to data limitation, we used children’s subjective reported closeness with parents to measure the parent–child relationship and the views of parents and other family members were neglected. Although this practice has been employed by previous studies (Videon, 2005; Damsgaard et al., 2014), a more objective way to reporting the parent–child relationship may be necessary for the future to ensure the validity of measurement.

## Conclusion

Our study observed an only child advantage in the parent–child emotional relationship. Only children were not only more favored compared to non-only children in general, they were also more favored in comparison with children from two-child families and firstborns of multiple-child families. Furthermore, we found that the sibship-size and birth-order effects were gender-specific: daughters benefited more from being only children. We also found the gender asymmetric sibling effects that daughters were disadvantaged by having younger brothers, whereas sons benefited more from having older sisters. Our findings highlighted the importance to consider children’s gender when exploring the only child effects.

A large body of literature has documented various developmental outcomes of only children. However, relatively limited research has focused on the family relationships of only children in the Chinese context. Our study contributed to the current knowledge of only children by exploring their parent–child relationships. In addition to comparing only children to children with siblings, we also took another step forward by exploring the birth-order effects and gender-composition effects. Our study has important policy implications. Policy-makers should be highly aware of the persistence of “valuing sons but devaluing daughters” culture in the Chinese multiple-child families and formulate some policies to weaken this idea, especially in the universal two-child policy era.

## Data Availability Statement

The datasets presented in this study can be found in online repositories. The names of the repository/repositories and accession number(s) can be found below: http://cnsda.ruc.edu.cn/index.php?r=projects/view&id=72810330.

## Ethics Statement

The studies involving human participants were reviewed and approved by the ethics committee of Renmin University of China. Written informed consent to participate in this study was provided by the participants’ legal guardian/next of kin.

## Author Contributions

YL designed the study, processed the data, and drafted the original manuscript. QJ provided the data and revised the manuscript. Both the authors critically reviewed and approved the final manuscript.

## Conflict of Interest

The authors declare that the research was conducted in the absence of any commercial or financial relationships that could be construed as a potential conflict of interest.
